# Workload, missed nursing care, ethical climate, and work interruptions among emergency department registered nurses: A descriptive-analytical study

**DOI:** 10.1016/j.ijnsa.2026.100562

**Published:** 2026-05-27

**Authors:** Sudabeh Esmaeili, Ali Mohammad Parviniannasab, Mostafa Bijani

**Affiliations:** a Student Research Committee, Fasa University of Medical Sciences, Fasa, Iran; b Department of Nursing, School of Nursing and Midwifery, Shiraz University of Medical Sciences, Shiraz, Iran; c Department of Medical Surgical Nursing, School of Nursing, Fasa University of Medical Sciences, Fasa, Iran

**Keywords:** Emergency department, Missed nursing care, Workload, Work interruption

## Abstract

**Background:**

Nurses working in emergency departments encounter a wide range of professional challenges stemming from the inherently complex, unpredictable, and high-intensity nature of emergency care. These challenges exert a considerable influence on their clinical performance. Among the most critical factors, workload and work interruptions are thought to negatively affect both the occurrence of missed nursing care and the ethical climate within the emergency departments. In light of these considerations, we sought to examine the relationship between workload and missed nursing care, with particular attention to the mediating roles of ethical climate and work interruptions among emergency department registered nurses.

**Methods:**

This descriptive-analytical study was conducted among 700 emergency registered nurses in southern Fars Province, Iran. Data were gathered using a demographic questionnaire, the Missed Nursing Care Questionnaire, the Hospital Ethical Climate Survey, the Nursing Work Interruption Scale, and the NASA Task Load Index. Structural equation modeling was used for analysis.

**Results:**

The participants’ mean age and mean work experience were 35.26 years (SD=6.80), and 8.71 years (SD=5.38), respectively. The pearson correlation analysis revealed positive associations between workload and missed nursing care was positive and statistically significant (*r* = 0.225, *p* < 0.01), and workload was negatively and significantly-related to ethical climate (*r*=-0.222, *p* < 0.01). In contrast, workload was significantly and positively correlated with work interruption (*r* = 0.199, *p* < 0.01). We found a negative correlation between ethical climate and work interruption (*r* = -0.168, *p* < 0.01), and work interruption was also strongly and positively associated with missed nursing care *(r* = 0.482, *p* < 0.01). Finally, ethical climate was negatively and statistically- significantly related to missed nursing care. Furthermore, the indirect effect of workload on missed nursing care, through the sequential mediation of ethical climate, and work interruption was also statistically significant (bootstrap 95% confidence interval 0.09, 0.034).

**Conclusion:**

We have revealed a significant positive relationship between workload, work interruptions, and missed nursing care among emergency registered nurses in Iran. We suggest that fostering a positive ethical climate within the emergency department may play a crucial role in reducing missed nursing care and improving overall care quality. Additionally, given that excessive workload and frequent interruptions were associated with both ethical climate and care delivery, it may be essential for nursing leadership to implement systematic and proactive strategies to address these stressors.

▓

**What is already known**

• Workload and work interruptions are thought to negatively affect both the occurrence of missed nursing care and the ethical climate within the emergency department.
• No prior study was identified that concurrently investigated the interrelationships among all four variables (workload, work interruptions, missed nursing care, and ethical climate) within the context of emergency department using structural equation modeling.

**What this paper adds**

• We have revealed a significant positive relationship among workload, work interruptions, and missed nursing care among emergency department nurses.
• We have provided evidence of an indirect effect of workload on missed nursing care through the sequential mediation of ethical climate and work interruption.
• We suggest that fostering a positive ethical climate within the emergency department may play a crucial role in reducing missed nursing care and improving overall care quality.

Alt-text: Unlabelled box dummy alt text

## 1 Introduction

Nurses in emergency departments work in environments marked by complexity, unpredictability, high patient turnover, and rapidly evolving clinical conditions (Trisyani et al., 2023). These demanding conditions pose substantial professional challenges that can undermine their performance and quality of care (Trisyani et al., 2023). Among the various occupational stressors, workload has been identified as one of the most critical factors affecting nurses’ professional functioning, the quality of care provided, and patient satisfaction within emergency care settings (Soola, Mehri, and Azizpour, 2022). Numerous researchers have consistently documented the prevalence of excessive workload among emergency department nurses (Brassington et al., 2025; Yusefi, Ebrahim, Jame, and Bastani, 2019), a condition that is closely linked to a wide array of adverse outcomes. These include a higher incidence of clinical errors, prolonged hospitalization, increased readmission rates, diminished quality of care, and compromised patient safety (Montazer Ataei, Karimi Monaghi, Ghavidel, and Grivani, 2021; Riu et al., 2024). Additionally, elevated workload levels contribute to psychological strain, fatigue, and burnout, which in turn increase the likelihood of nurse attrition and turnover (Barzani and Dal Yılmaz, 2022; Galanis et al., 2025). Researchers have suggested that excessive workload is a major contributing factor to the phenomenon of missed nursing care (Amiri Amjad, Jahani, Sayadi, and Cheraghian, 2023; Tubbs-Cooley, Mara, Carle, Mark, and Pickler, 2019). Given the inherent urgency and clinical intensity of emergency departments, some degree of missed care may be unavoidable, and contributing factors include patient acuity, understaffing, excessive workload, limited access to medical equipment, sudden surges in admissions, and general overcrowding, each of which significantly increases the risk of care omissions in these settings (Chegini et al., 2020; Hanifi, Eskandari, Sharif, and Rahmatpour, 2025). Work interruptions refer to task suspensions, delays,or forced task switching caused by internal or external disruptions during work processes (Yu and Lee., 2022). Shan et al. (2023) documented that prolonged exposure to such interruptions may reduce nurses’ vigilance and capacity to respond in complex situations. Danesh et al., 2022, documented the high frequency of work interruptions in emergency departments, attributing this trend to elevated nurse workloads and overcrowding. In the nursing context, ethical climate is nurses’ subjective evaluations of how ethical challenges are managed within their workplaces. Given that nursing practice is embedded within a complex social matrix of institutional structures and interpersonal dynamics, the prevailing ethical atmosphere exerts a profound influence on nurses’ moral reasoning, behavior, and overall delivery of care (Koskenvuori, Numminen and Suhonen, 2019). A positive ethical climate in hospitals reduces feelings of isolation and is associated with improved performance and patient satisfaction (Ozdoba et al., 2022). Additionally, as positive ethical climate improves job satisfaction, decreasing turnover and nursing shortages (Ozdoba et al., 2022). Wang et al., 2022, highlighted that nursing managers, by fostering a more constructive ethical climate, can promote ethical conduct, support professional development, and simultaneously reduce both workload and missed care, thereby enhancing the overall safety and quality of nursing services.

## 2 Research gap and significance of the study

Despite numerous studies exploring workload, work interruptions, missed nursing care, and the influence of ethical climate, these variables have typically been examined in isolation. In some instances, researchers have addressed the relationships between two of these constructs or focused exclusively on the effect of ethical climate on a single variable. However, despite extensive and systematic database searches, no prior study was identified that concurrently investigated the interrelationships among all four variables—workload, work interruptions, missed nursing care, and ethical climate—within the context of emergency departments using structural equation modeling (SEM). SEM is a powerful tool for mediation analysis (Sim, Kim and Suh., 2022). Therefore, considering the importance of the subject and also to fill the research gap and develop nursing knowledge, we aimed to examine the relationship between workload and missed nursing care, with ethical climate and work interruption serving as sequential mediators among emergency department nurses. Accordingly, we proposed the following hypotheses: 1. Workload is significantly associated with missed nursing care among emergency department nurses. 2. Ethical climate and work interruption sequentially mediate the relationship between workload and missed nursing care among emergency department nurses.

## 3 Methods

A descriptive-analytical cross-sectional design was employed for this study, which was conducted from May 2025 to August 2025 and targeted a population of emergency department nurses.

### 3.1 Sampling and data collection process

The research sample consisted of registered nurses (RNs) working in the emergency units of the five largest teaching hospitals affiliated with academic medical centers in Fars Province, located in southern Iran, all of the which operate fully-functional and specialized emergency departments. Within these settings, RNs working in the emergency units provide comprehensive nursing care to a diverse and broad spectrum of patients seeking emergency services. Participants were selected through a non-probability convenience sampling method. Inclusion criteria required nurses to be employed full-time an emergency departments, to have at least 1 year of clinical experience, and to voluntarily consent to participate in the study. The minimum required sample size was determined based on the number of questionnaire items, applying a participant-to-item ratio ranging from 5 to 10 (Sim, Kim and Suh., 2022). Given the total of 68 items across all instruments, the upper threshold for sample size was estimated at 680 participants. To account for potential attrition due to nonresponse or incomplete data, a 10% oversampling adjustment was incorporated, resulting in a target sample size of 748. Ultimately, 748 questionnaires were distributed; of these, 700 were returned fully completed and included in the final analysis (Hospital 1: *n* *=* 152, Hospital 2: *n* = 142, Hospital 3: *n* = 139, Hospital 4: *n* = 132, Hospital 5: *n* = 135). Thus, the response rate (93.58%) was acceptable. Data collection was conducted using a systematic, stepwise approach. After obtaining all required institutional approvals, scheduled visits to the designated hospital wards were arranged to coincide with the start of RN shifts. RNs who met the eligibility criteria were thoroughly informed about the study objectives, the overall research design, and the specific procedures for completing the questionnaire. They were instructed to complete the questionnaire independently and to submit the completed form to the head nurse of their respective ward. To ensure consistency in implementation and to reduce the likelihood of misunderstanding, head nurses received comprehensive instructions regarding questionnaire administration. Head nurses and the first author were responsible for addressing any questions raised by participating RNs. The researcher met with the participants in a quiet area for 20 to 30 min during breaks and between shifts and 1 week after distribution, the research team returned to retrieve the completed questionnaires from the head nurses.

### 3.2 Data collection instruments

#### 3.2.1 Demographic information questionnaire

The demographic information questionnaire included questions about age, sex, work experience, education level, and marital status.

#### 3.2.2 Missed nursing care questionnaire

The Missed Nursing Care Questionnaire, originally developed and validated by Kalisch and Williams (2009), consists of 24 items rated on a 4-point Likert scale: “Rarely missed” (score of 1), “Occasionally missed” (score of 2), “Frequently missed” (score of 3), and “Always missed” (score of 4). The total score ranges from a minimum of 24 to a maximum of 96, with higher scores indicating a greater frequency of missed nursing care. The Persian version of this instrument was psychometrically evaluated by Yaghoubi et al., 2019, using a rigorous forward–backward translation method to ensure linguistic and conceptual fidelity. Construct validity was assessed through both content and face validation by a panel of subject matter experts who reviewed item clarity, relevance, and cultural appropriateness. Based on expert feedback, necessary modifications were incorporated to finalize a contextually valid version. Internal consistency reliability was calculated with a Cronbach’s alpha of 0.92. In their study in Iran, Najafi Ghezeljeh et al. (2021) calculated the reliability coefficient at α = 0.89. In the present study, the reliability coefficient was calculated at α=0.89, further supporting the instrument’s psychometric robustness for the target population.

### 3.3 Hospital ethical climate survey

The Hospital Ethical Climate Survey, originally developed by Olson in, 1995 in the United States, comprises 26 items that assess healthcare professionals’ perceptions of the ethical climate within their hospital units. Responses are recorded on a 5-point Likert scale ranging from “Never” (1) to “Almost always” (5). Total scores range from 26 to 130, with scores of 78 or higher indicating a satisfactory ethical climate. Olson reported high internal consistency in the original validation, with a Cronbach’s alpha of 0.91. The first Persian-language adaptation of the Hospital Ethical Climate Survey was conducted by Mobasher et al. (2008), who reported excellent internal consistency (Cronbach’s α = 0.92). In another study in Iran, the reliability of the instrument was confirmed with a Cronbach’s alpha of 0.87, indicating strong internal consistency and reliable performance across the sample (Mobasher et al., 2008). In the present study, the reliability coefficient was calculated at α=0.88, showing that the internal consistency of this questionnaire was at a good level.

### 3.4 Nursing work interruption scale

The Nursing Work Interruption Scale was developed and validated by Yu and Lee (2022). This instrument includes 12 items rated on a 6-point Likert scale ranging from “At least 5 times per day” (score of 6) to “Almost never” (score of 1). Total scores range from 12 to 72, with higher scores reflecting a greater frequency of work interruptions. In their original study, the internal consistency was validated with a Cronbach’s alpha of 0.87 (Yu and Lee, 2022). The Nursing Work Interruption Scale was subsequently translated into Persian and validated by Jalali et al., 2025. Confirmatory factor analysis (CFA) supported the scale’s construct validity, yielding strong model fit indices: comparative fit index (CFI) = 0.91, non-normed fit index (NNFI) = 0.92, goodness of fit index (GFI) = 0.91, root mean square error of approximation (RMSEA) = 0.057, and standardized root mean square residual (SRMR) = 0.045. Reliability was further substantiated by a Cronbach’s alpha of 0.896 and McDonald’s omega of 0.892, confirming the instrument’s strong psychometric properties in the local context (Jalali et al., 2025). In the present study, the reliability coefficient was calculated at α=0.89, indicating that the internal consistency of this questionnaire was at a good level.

### 3.5 NASA task load index

The NASA Task Load Index provides the most widely accepted and validated tool to measure overall workload after completing a task. The NASA Task Load Index contains six dimensions (1) mental demand, (2) physical demand, (3) temporal demand, (4) effort, (5) performance (efficacy), and (6) frustration level. Each dimension is rated on a scale from 0 to 100. A mean score below 50 is considered indicative of an acceptable workload, where as a score ≥50 signifies a high workload (Hart and Staveland, 1988). The content and face validity of the Persian version of the NASA Task Load Index was evaluated and confirmed by Yusefi et al., 2019, through a structured assessment with Iranian nursing professionals. Internal consistency was established with a Cronbach’s alpha of 0.90, affirming the instrument’s reliability in the target population. In the present study, the reliability coefficient was calculated at α = 0.87, indicating that the internal consistency of this questionnaire was at a good level.

### 3.6 Data analysis

To examine the interrelationships among the study variables, Pearson correlation coefficients were calculated using IBM SPSS Statistics, version 27. To assess the construct validity of the proposed measurement model, CFA was conducted. Model fit was evaluated using a comprehensive set of indices, including the chi-square to degrees of freedom ratio (χ²/df), RMSEA, SRMR, GFI, CFI, and Tucker–Lewis Index (TLI). Acceptable model fit was defined by the following thresholds: χ²/df <5.00, RMSEA and SRMR values below 0.08, and GFI, CFI, and TLI values exceeding 0.90 (Sim, Kim and Suh., 2022). The model was a good fit to the data with a χ2/df = 189.52/44 = 4.30 < 5, GFI = 0.932, CFI = 0.944, TLI = 0.963, RMSEA = 0.079, and SRMR=0.071. To assess the hypothesized mediation model, demographic covariates were statistically controlled. None of these variables demonstrated a significant predictive effect on the dependent variable. The mediating roles sequential mediation analysis of ethical climate and work interruption in relationship between workload and missed nursing care performance were tested using Hayes’ PROCESS macro, version 4.2 (Model 6). Indirect effects were estimated via bootstrapping with 5000 resamples, and statistical significance was determined at a conventional alpha level of *p* < 0.05.

### 3.7 Ethical considerations

We conducted the study in full accordance with the principles outlined in the revised Declaration of Helsinki, which serves as the internationally-recognized ethical framework for biomedical research involving human participants. Prior to data collection, all participants were provided with a clear and concise explanation of the study's objectives. Subsequently, they completed a brief demographic questionnaire. In line with strict confidentiality protocols, no personally identifiable information was collected. Informed consent was implied through the voluntary completion of the questionnaire, which also emphasized participants’ right to withdraw at any point. Ethical approval for the research was granted by the Institutional Research Ethics Committee of Fasa University of Medical Sciences, Fasa, Iran (Approval Code: IR.FUMS.REC.1404.047).

## 4 Results

### 4.1 Sample characteristics

The participants’ mean age and mean work experience were 35.26 ± 6.80 and 8.71±5.38 years, respectively. The personal characteristics of the participants are shown in Table 1.

**Table 1:** Demographic characteristics of study participants (*N=700*).

Variables	*n* (%)
Sex	
Male	294 (42)
Female	406 (58)
Education	
Bachelor's degree	665 (95)
Master's	35(5)
Marital status	
Single	245 (35)
Married	455 (65)
Work experience (Years)	
≤5	60 (5.5)
6–10	285 (40.71)
>10	355 (50.72)

### 4.2 Correlational findings

The correlation between workload and missed nursing care was positive and statistically significant, and workload was negatively and significantly related to ethical climate. In contrast, workload was significantly and positively correlated with work interruption. Moreover, ethical climate demonstrated a negative correlation with work interruption, and work interruption was also strongly and positively associated with missed nursing care. Finally, ethical climate was negatively and significantly related to missed nursing care. (Table 2).

**Table 2:** Means, standard deviations, and correlations among study variables.

Variable	M	SD	1	2	3	4
1. Missed nursing care	70.24	13.14	1			
2. Ethical climate	99.48	27.27	−0.369⁎⁎	1		
3. Work interruption	55.73	7.62	0.482⁎⁎	−0.168⁎⁎	1	
4. Workload	63.96	7.60	0.255⁎⁎	−0.222⁎⁎	0.199⁎⁎	1

Note: M: mean; SD: standard deviation.

⁎⁎ indicates significance at *p* < 0.01 level.

### 4.3 Mediation analyses

**Fig. 1:**
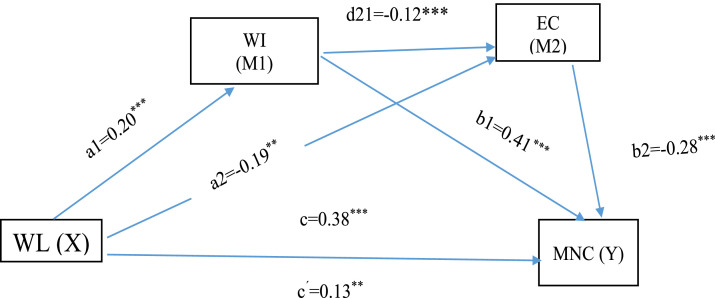
Model illustrating the sequential mesdiating effects of ethical climate and work interruption in relationship between workload and missed nursing care. Note: MNC: Missed nursing care; EC: Ethical climate; WI: Work interruption; WL: Workload. Standardized path coefficients are shown. *** indicates significance at *p<* 0.001 level.

We used skewness and kurtosis values with an accepted range of −2 to +2 (Sim, Kim and Suh., 2022) to test the normality. Workload, ethical climate, and work interruption collectively explained 33% of the variance in missed nursing care. Furthermore, the model supported the mediation hypothesis. Although the default PROCESS macro specifies two sequential mediating variables, the statistical analysis approach based on Hayes’ Model 6 defines one independent variable, two mediators, and one dependent variable. It should be noted that each mediating variable can also function as an independent variable for the subsequent dependent variable in the model. Accordingly, we attempted to rearrange the positions of ethical climate and work interruptions so that work interruptions would serve as an independent variable for both ethical climate and missed care. In accordance with our hypotheses, the indirect effect of workload on missed nursing care through the sequential mediation of ethical climate and work interruption (Tables 3 and Fig. 1).

**Table 3:** Standardized total, direct, and indirect effects of workload on missed nursing care mediated by and work interruption ethical climate.

Path	Unstd.	Std.	SE	t-value	p	R^2^	LLCI	ULCI
WL→WI	0.19	0.20	0.03	5.29	0.000^⁎⁎⁎^	0.039	0.12	0.27
WL→EC	−0.70	−0.19	0.13	−5.18	0.000***	0.060	−0.97	−0.43
WI→EC	−0.46	−0.12	0.13	−3.39	0.0007**		−0.72	−0.19
WL→MNC	0.13	0.07	0.05	2.41	0.015**	0.32	0.02	0.24
WI→MNC	0.72	0.41	0.05	12.78	0.000***		0.61	0.83
EC→MNC	−0.13	−0.28	−0.01	−8.57	0.000***		0.16	0.10
Indirect effect								
WL→WI→MNC	0.14	0.08	0.03				0.07	0.21
WL→EC→MNC	0.09	0.05	0.02				0.05	0.13
WL→WI→EC→MNC	0.010	0.007	0.004				0.004	0.02
Total effect								
WL→MNC	0.38	0.22	0.06	6.02	0.000*	0.050	0.26	0.51

SE: standard error; Unstd.: unstandardized coefficients, Std: standardized coefficients, LLCI: lower limit confidence interval, ULCI: upper limit confidence interval, *p*-value –probability of result occurring by chance. R²: Variance explained by independent variables, t-value: Test statistic for significance. MNC: Missed nursing care; EC: Ethical climate; WI: Work interruption; WL: Workload. indicates significance at *p < 0.001, **p < 0.05.

## 5 Discussion

We aimed to examine the relationship between workload and missed nursing care, with ethical climate and work interruption serving as sequential mediators among emergency department RNs. We found a direct and positive relationship between work interruptions, workload, and missed nursing care. Specifically, increased frequency of work interruptions was associated with higher perceived workload and a greater incidence of missed nursing care. These results are consistent with prior researchers, who have reported similar associations (Abere, Biresaw, Misganaw, Netsere, and Adal, 2024; Alsalem et al., 2023; Gong, Mei, Wu, and Tang, 2025). Accordingly, it may be essential for nursing administrators to implement targeted interventions aimed at identifying and minimizing sources of work interruptions in emergency settings. Such interventions may help reduce nurses’workload and subsequently lower the frequency of missed care. Furthermore, a significant inverse association was identified between ethical climate and the variables of work interruptions, workload, and missed nursing care. This finding is similar to the results of earlier studies conducted by Amini, Lehdarboni and Hanifi 2022; Noh, et al., 2023, and Dehghani et al., 2023, who reported a negative correlation between ethical climate and missed nursing care. However, in contrast, Abdrbo and Bayoumy, 2022, reported a direct and positive association between ethical climate and missed nursing care, which diverges from the outcomes we found. To interpret this discrepancy, we posit that, in environments where ethical principles are highly emphasized, nurses may devote more time to addressing ethical concerns and engaging in moral deliberation. While this focus is commendable, it may inadvertently result in the postponement or omission of certain routine care activities. A positive correlation between ethical climate and missed nursing care might, therefore reflect the complex interplay between moral responsibility and practical workload management. Nonetheless, despite the observed association, correlation does not imply causation. A correlational relationship simply denotes that changes in one variable are accompanied by changes in another, without necessarily indicating a direct causal link. The combination of workload, ethical climate, and work interruptions accounted for 33% of the variance in missed nursing care. In support of the proposed model, the results confirmed the sequential mediation hypothesis, demonstrating that workload influenced missed care indirectly through its effects on ethical climate and work interruptions.

Liu et al., 2025, also identified work interruptions as a mediator in the relationship between missed nursing care and overall nursing performance.Their findings reinforced the notion that interruptions in clinical workflows not only exert a direct impact on nurses’ physical and psychological well-being but also produce indirect effects by increasing perceived workload and the likelihood of care omissions. As such, it may be imperative to implement comprehensive strategies aimed at optimizing workflow efficiency, reducing the frequency of interruptions, and alleviating workload pressures and, thereby, minimizing the occurrence of missed nursing care.

### 5.1 Study limitations

Despite offering valuable insights, this study is subject to several methodological limitations. The use of a convenience sampling approach may have introduced sampling bias. To mitigate this, participants were recruited across multiple shifts and days to ensure sample heterogeneity and enhance representativeness. Nevertheless, the reliance on self-reported data introduces the possibility of response biases, such as social desirability or acquiescence bias, which may affect the validity of the findings. Additionally, the cross-sectional design limits the ability to draw causal inferences or observe temporal changes in the studied relationships. Longitudinal designs are needed to examine these dynamics over time. Moreover, the research was conducted exclusively in public hospitals in southern Iran, which may constrain the generalizability of the findings. Future research is recommended in private-sector institutions and in diverse international settings to improve external validity.

### 5.2 Strengths of the study

Among the notable strengths of this study are its relatively large and diverse sample, which increased statistical power and the precision of the estimates. Furthermore, the use of psychometrically-validated instruments with demonstrated reliability and validity enhanced the methodological rigor and credibility of the study's findings.

### 5.3 Implications for nursing management

We have provided potentially important guidance for improving emergency department nurses' health. Optimizing workflow and reducing work interruptions may effectively reduce nurses' perceived workload, thereby decreasing the occurrence of missed nursing care and ultimately contributing to the overall improvement of ethical climate and patient care. Nursing managers may need to implement targeted strategies aimed at addressing workplace challenges, particularly high workload and frequent disruptions. By doing so, they may be able to reduce the occurrence of missed nursing care and, consequently, enhance both the quality and safety of patient outcomes.

## 6 Conclusion

We found an inverse association between work interruptions, workload, and missed nursing care on one side and ethical climate on the other. We suggest that fostering a positive ethical climate within the emergency department may play a crucial role in reducing missed nursing care and improving overall care quality in Iran. Additionally, given that excessive workload and frequent interruptions can adversely impact both ethical climate and care delivery, it may be essential for nursing leadership to implement systematic and proactive strategies to address these stressors. By reducing workload and minimizing interruptions, nursing administrators may not only lower the incidence of missed care but also cultivate a more ethically-supportive clinical environment.

## Funding sources

This research received no specific grant from any funding agency in the public, commercial, or not-for-profit sectors.

## Data availability statement

The data that support the findings of this study are available from the corresponding author upon reasonable request.

## CRediT authorship contribution statement

**Sudabeh Esmaeili:** Methodology, Investigation, Data curation, Conceptualization. **Ali Mohammad Parviniannasab:** Writing – original draft, Software, Methodology, Investigation, Formal analysis, Conceptualization. **Mostafa Bijani:** Writing – review & editing, Writing – original draft, Project administration, Methodology, Investigation, Formal analysis, Conceptualization.

## Declaration of competing interest

The authors declare that they have no known competing financial interests or personal relationships that could have appeared to influence the work reported in this paper.
